# A Century Spent Combating Rabies in Morocco (1911–2015): How Much Longer?

**DOI:** 10.3389/fvets.2017.00078

**Published:** 2017-06-02

**Authors:** Sami Darkaoui, Florence Cliquet, Marine Wasniewski, Emmanuelle Robardet, Nadia Aboulfidaa, Mohammed Bouslikhane, Ouafaa Fassi-Fihri

**Affiliations:** ^1^Division of Pharmacy and Veterinary Inputs, National Food Safety Office, Rabat, Morocco; ^2^ANSES – Nancy Laboratory for Rabies and Wildlife, French Agency for Food, Environmental and Occupational Health & Safety, European Union Reference Laboratory for Rabies, WHO Collaborating Centre for Research and Management in Zoonoses Control, OIE Reference Laboratory for Rabies, European Union Reference Laboratory for Rabies Serology, Technopôle agricole et vétérinaire de Pixérécourt, Malzéville, France; ^3^Department of Pathology and Veterinary Public Health, Agronomic and Veterinary Institute Hassan II, Rabat, Morocco

**Keywords:** rabies, Morocco, epidemiology, dog, one health, vaccination coverage, dog population management

## Abstract

Rabies has no known beginning in Morocco and to date, government control efforts and plans fail to eradicate the disease. A review and analysis of available epidemiological data are crucial to learn lessons from the past and to propose effective actions. Legally, animal rabies is a notifiable disease since 1913 and legislation has been updated periodically since. Dogs have always been considered as both the disease’s vector and reservoir, while cattle, other herbivores, and humans are victims. Animal rabies cases evolution from 1942 to 2015 is characterized by ascending phase then decreasing one following structured rabies control plan implementation in 1980s. Indeed, from 1986 to 2010, three rabies control plans have been conducted based on free of charge rabies vaccination of owned dogs through mass campaigns. The geographical distribution of rabies is stable over the years with highest cases number in rich rural areas and around cities. Human rabies cases are decreasing over the time (1976–2015) thanks to the opening of new antirabic treatment centers in the last decade which permit the administration of more PEPs. After a century of rabies control, Morocco registered an average of 301 animal cases and 21 human cases annually for the last decade (2005–2015). Few reasons led to those limited results. The lack in law enforcement and, moreover, the fact that the law do not take into account responsible dog ownership aspect are of importance. Lack of dog population knowledge and management and intersectoral coordination deficiency are additional failure reasons. The gathered data will help to build a new strategy with a focus on a “One Health” approach. Dog population ecology parameters’ study is of primary importance. We estimated dog population to be 2.8 million dogs based on human:dog ratio. Enhancing vaccination coverage of dog population is feasible by combining parenteral vaccination and complementary oral vaccination. Updating legislation by inclusion of responsible dog ownership and law enforcement are crucial. Over the last century, Morocco registered a slow decreasing tendency in the number of animal and human rabies cases. Urgent strategy need to be implemented because rabies elimination is an achievable goal in Morocco.

## Introduction

Rabies has no known beginning or starting point in Morocco (1). Several epidemiological studies have focused on this disease since the beginning of rabies vaccination among dogs in 1927 (2). They have provided a wealth of documentation covering a century of rabies surveillance in Morocco. Compilation and analysis of data are of importance to learn lessons from the past and to propose actions to be included in any new strategy.

Efforts to control rabies in Morocco began as early as 1911, year of the first human and canine rabies vaccination by a local vaccine manufactured at Institute Pasteur of Tangier (IPT) (1). Since that, routine antirabies vaccination of people and animals was practiced but without any improvement in the number of victims. The World Health Organization first launched initiatives to set up a rabies control program, supported by international experts, in 1980s (3–5). The program had an objective of rabies elimination through a certain number of actions related to human health, veterinary sector, and municipalities. The limited results led to other control strategies in 1990 and 2001. Despite these efforts, Morocco still recorded an average of 21 human rabies cases per year (2005–2015) (6) and since 1923, official documents have recorded the death of over 1,046 humans due to rabies and the exposure of over 838,660. The number of people having been given postexposure prophylaxis (PEP) is enough to fill a big-sized town such as Sale (city in north-west of Morocco, has a population of 850,403 according to the census of 2014, and is the fifth biggest city of country).

The rabies situation in Morocco affects not only Moroccan citizens but also neighboring countries. The density of trade with Europe exchanged by road is a source of rabies contamination for several rabies-free European countries. A number of cat and/or dog owners illegally smuggle their pet(s) into Europe mostly by road through Spain, disregarding all the legal provisions concerning the transportation of animals into Europe and not declaring their animal(s) to the customs officials or veterinary border control staff (7–10). From 2001 to 2015, 12 rabies alerts have been notified in Europe originating from Morocco (eight in France, one in Belgium, one in Germany, one in the Netherlands, and one in Spain) (10).

An analysis of the experience of the past century should reveal lines of action in order to make better headway; future strategies should also be able to benefit from scientific and technical progress in the field of rabies control (11). Morocco is a developing country and should choose the most efficient means of control in order to reduce the costs incurred by rabies (12) and eliminate the risk of exposing the Moroccan population to this fully preventable disease.

## Materials and Methods

### Rabies Regulations

A review of the regulatory texts on rabies published between 1913 and 2014 in Moroccan official journal^1^ is realized with focus on the legal status of rabies. The key points of each text are presented and general evolution of legislation related to rabies is discussed.

### Epidemiological Analysis

Epidemiological data concerning animal and human rabies cases were collected from published bibliographical references and epidemiological reports issued by the competent public authorities, and more especially those of Morocco’s National Food Safety Office (ONSSA) for animal rabies cases and the Ministry of Health for human cases. In the event of contradictory data from different sources, the highest figure has been chosen due to the substantial risk of underreporting (13). The epidemiological characteristics of animal rabies in Morocco are discussed for the period from 1928 to 2015. There follows a presentation and discussion of the rabies patterns in Morocco (reservoir, animal species affected, seasonal changes, and geographical distribution) as well as measures taken at different times to tackle the disease and the suggestions of various authors in order to improve the situation. Mapping of the evolution of the number of animal rabies cases in the Moroccan provinces was performed using ArcGIS 10.1 software. The number of human rabies cases reported annually from 1974 to 2015 is analyzed and discussed. There is a discussion on the geographical distribution of cases of human rabies. The provided PEP protocol is discussed as well as the laboratory diagnosis. Propositions of improvement are formulated. A review of the genetic characterization of rabies virus isolates in Morocco is realized. Molecular epidemiology data are compared with classical epidemiology findings.

### National Rabies Control Plans (NRCPs)

Different NRCPs are presented. The achievements and deficiencies of plans are presented and the causes of their limited success analyzed.

### Moroccan Dog Population

It is crucial to remain informed about the dog population in order to draw up a realistic and effective canine rabies control plan. A synthesis of the studies that are interested in the size of the Moroccan canine population is carried out in order to have an estimate of this population. This is based on a comparison between the results of estimations of the dog population in the framework of an amendment to the 1993 rabies control plan, the NRCP of 2001–2010 and other international studies or studies in Maghreb countries. The data are extrapolated depending on the human:dog ratio chosen in order to estimate the size of the dog population. Demographic data were provided by official Moroccan authorities (Health Ministry website: http://www.sante.gov.ma). The current state of dog population control and suggestions for improvement are also presented.

### Vaccination Coverage of the Dog Population

The vaccination coverage of the dog population is estimated by combining data on vaccination in urban areas by private veterinarians with that in rural areas following government-run mass vaccination campaigns. Dog population data are related to the estimated human:dog ratio. Vaccination coverage is analyzed and suggestions put forward on how to improve the situation. Data on vaccination in urban areas were extrapolated from the number of doses of rabies vaccine sold between 2009 and 2015 to veterinarians in the private sector. The data were obtained from veterinary pharmaceutical companies with a rabies vaccine registered in Morocco^2^ during the same period. Propositions for dog population vaccination coverage amelioration are formulated with reference to Moroccan experiments in oral dog rabies vaccination.

### Application of the “One World, One Health” Concept

The “One World, One Health” concept is highlighted by international organizations such as FAO, OIE, and WHO as the most efficient way of controlling zoonotic diseases. The actions taken in Morocco are assessed in the light of this concept, examples, and suggestions given.

## Results and Discussion

### Rabies Regulations in Morocco

Morocco has a wealth of regulations on animal rabies control with a new text every 9 years. These regulations were first focused in people treatment after rabies exposition on IPT (14) and at antirabies institute of Marie-Feuillet hospital in Rabat (15). The measures were also concentrated on prevention of exposure to rabid animals either imported (16) or locally by instigating measures (17) to control stray dogs in both urban settings (18) and the countryside (19). Parenteral vaccination of dogs was already mandatory in 1928 (20) early and quickly after OIE 1927 conference on rabies and mandatory antirabies vaccination of cats was instaured on 1934 (21).

This basic regulatory framework has been updated in several occasions either within mandatory notifiable animal diseases list update in 1977 (22) or by updating specific measures to control rabies in 2000, 2005, and 2014 (23–25).

This regulation evolves from the vizirial orders in 1915, 1927, 1928, 1934, 1936, to decrees in 2000, 2005, and 2014, which have more regulatory weight.

The regulations cover mandatory rabies vaccination of dogs and measures to be taken in the event of contamination but they do not cover management of the dog population or the concept and actions of “responsible dog ownership”. The latter concept implies registering and identifying dogs, preventing their negative impact on society and controlling breeding. The legislation should also cover mass control actions for stray dogs (means of capture and humane euthanasia) in accordance with OIE rules on animal welfare (26).

It should be noted that national regulations are not fully applied. The mandatory vaccination of dogs is not monitored and there are no legal sanctions should a dog be found to be unvaccinated (25).

### Epidemiological Analysis

The first rabies vaccination was performed in Morocco in 1911 by a rabies vaccine for human and veterinary use produced at IPT (1, 27). Since that, exposed people were treated and from 1923 to 1932 a total of 361 people received PEP in IPT (21). The number of exposed treated people increased to reach an average of 1,500 person per year between 1951 and 1958 (28).

Veterinary authority started dogs preventive antirabies vaccination in 1928 and an average of 671 dogs per year were vaccinated between 1928 and 1933 (27, 29). This number increased to reach 10,000 dogs per year between 1945 and 1965 (30).

An average of 302 (minimum 68 and maximum 663) animal rabies cases was registered between 1942 and 1968 (28, 31–33) demonstrating an active animal rabies surveillance system based on Casablanca laboratory.

**Figure 1:**
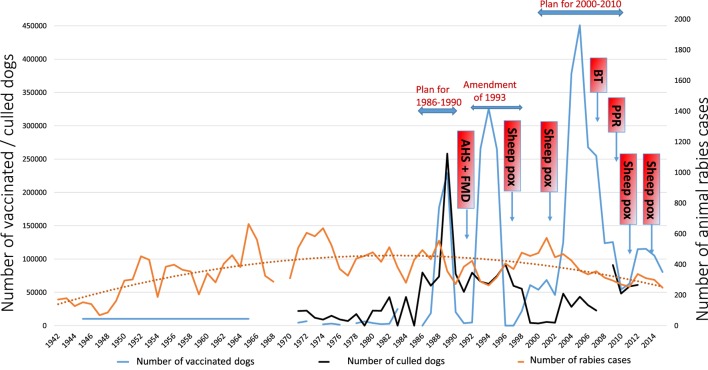
**Evolution of animal rabies cases, number of vaccinated and culled dogs (1942–2015)**. AHS, African Horse Sickness; FMD, Foot and Mouth Disease; BT, Bluetongue disease; PPR, Peste des petits ruminants disease.

Table 1 summarizes the data relating to the 1971 to 2015 period concerning animal rabies, the number of dogs vaccinated and culled, the number of human cases, and the number of people given PEP. We can identify an increasing trend of animal rabies cases number up to 1982 from where this trend decreases. This can be visualized in Figure 1, which traces evolution of animal rabies cases, number of vaccinated and culled dogs from 1942 to 2015. The animal rabies cases number follows a distribution with an ascending phase in which the number of animal rabies cases increases and a descending phase from 1982. The year 1982 coincides with start of WHO rabies fight in Maghreb zone (3). Those efforts led to first NRCP (1986–1990) launch. Figure 1 shows also that the number of vaccinated dogs per year increases considerably in the beginning of the control plan rising from 2,730 dogs in 1982 to 25,000 dogs in 1983 and 229,231 dogs in 1989. This figure then dwindled before increasing once again during the amended rabies control plan of 1994, reaching 325,780 dogs. Similarly, a peak of 450,917 dogs vaccinated was reached in 2005 under the 2001–2010 rabies control program. This evolution in the number of vaccinated dogs in peaks during the maximal activity of different rabies control plans is characteristic. The decrease in the number of vaccinated dogs is thought to be linked to the veterinary services efforts to control other livestock diseases (Figure 1) (5, 34).

**Table 1:** **Number of animal rabies cases, number of vaccinated dogs and culled dogs, number of PEPs, and number of human rabies cases from 1971 to 2015**.

Year	Number of animal rabies cases (reference)	Number vaccinated dogs (reference)	Number of culled dogs (reference)	Number of PEPs (reference)	Number of human rabies cases (reference)
1971	508 (35)^a^	4,492 (35)	21,952 (35)	9,011 (36)	

1972	606 (35)^a^	6,524 (35)	22,477 (35)	9,045 (36)	

1973	583 (35)^a^	ND (35)	11,676 (35)	10,209 (36)	

1974	635 (35)^a^	1,866 (35)	8,924 (35)		67 (4)

1975	525 (35)^a^	3,095 (35)	14,671 (35)		12 (4)

1976	370 (37)	1,303 (35)	9,379 (35)		14 (4)

1977	326 (35)^a^	ND (35)	6,788 (38)	11,109 (38)	15 (4)

1978	438 (37)^a^	3,511 (35)	17,375 (35)	15,769 (38)	50 (38)

1979	455 (37)^a^	6,250 (35)	28,502 (38)^a^	16,789 (38)	38 (4)

1980	479 (35)^a^	3,700 (35)	22,478 (38)	16,557 (38)	50 (4)

1981	417 (35)^a^	2,106 (35)	22,273 (38)	16,123 (38)	52 (39)^a^

1982	512 (35)	2,730 (35)	42,660 (38)	16,822 (38)	25 (39)^a^

1983	381 (37)	25,000 (3)	42,928 (38)	14,549 (38)	15 (39)^a^

1984	280 (40)		43,000 (3)^a^	14,960 (38)^a^	20 (39)^a^

1985	430 (40)		50,989 (38)	15,441 (38)	34 (4)^a^

1986	493 (5)	0 (5)	79,580 (38)^a^	15,954 (38)	34 (38)^a^

1987	433 (5)	18,569 (5)	59,958 (38)^a^	14,678 (41)	28 (4)^a^

1988	555 (5)	176,981 (5)	73,570 (5)	16,704 (41)	27 (4)^a^

1989	356 (5)	229,231 (5)	258,224 (5)	12,905 (41)	17 (39)^a^

1990	271 (5)	20,355 (5)	77,767 (5)	11,660 (41)	16 (39)

1991	384 (5)	3,527 (5)	50,666 (5)	10,496 (41)	18 (39)

1992	423 (5)	4,785 (5)	79,433 (5)	15,204 (41)	20 (39)

1993	287 (5)	265,731 (5)	65,986 (5)	11,562 (41)	24 (39)

1994	263 (5)	325,780 (5)	62,599 (5)	12,636 (41)	16 (39)

1995	317 (5)	264,739 (5)	74,425 (5)	14,700 (41)	29 (39)

1996	406 (5)	0 (5)	92,356 (5)	13,035 (41)	18 (39)

1997	369 (5)	0 (5)	59,562 (5)	13,906 (42)	21 (39)

1998	476 (5)	21,729 (5)	55,224 (5)	14,726 (42)	20 (5)^a^

1999	455^b^	60,839 (43)	4,145 (43)	13,742 (44)	30^a,b^

2000	474^b^	53,941 (43)	3,153 (43)	13,539 (45)	15^b^

2001	572^b^	68,548 (43)	5,470 (43)	14,196 (46)	26^b^

2002	446^b^	45,976 (43)	4,317 (43)	15,188 (47)	23^b^

2003	467^b^	124,688 (43)	47,799 (43)	15,425 (48)	17^b^

2004	425^b^	378,519 (43)	28,102 (43)	19,529^c^	23^a,b^

2005	360^b^	450,917 (43)	43,125 (43)	23,564^c^	25^b^

2006	335^b^	267,794 (43)	30,646 (43)	25,857^c^	16^b^

2007	355^b^	254,753 (43)	22,614 (43)	29,580^c^	31^b^

2008	313^b^	123,612 (49)		32,214^c^	24^b^

2009	294^b^	125,495 (49)	90,870 (49)	30,350^c^	17^b^

2010	269^b^	54,172 (50)	48,288 (50)	28,097^c^	19^a,b^

2011	253^b^	62,851 (51)	58,867 (51)	27,885^c^	18^b^

2012	337^b^	114,790 (52)	61,006 (52)	30,365^c^	19^b^

2013	310^b^	115,274 (53)		32,692^c^	24^b^

2014	299^b^	105,181 (54)		35,279^c^	20^b^

2015	248^b^	80,613 (55)		32,547^c^	19^b^

*^a^Discordant data*.

*^b^Data provided by the ONSSA/DSV Epidemiology and Health Monitoring Service*.

*^c^Data provided by the Epidemiology and Disease Control Directorate of the Moroccan Ministry of Health*.

*ND, not determined*.

*Blue color: data for the period before implementing the first NRCP*.

*Orange color: data for the period after implementing the first NRCP*.

Up to 1992, the number of dogs culled was always greater than the number of dogs vaccinated. Since 1992, the culling of dogs has been stopped in the context of rabies control but is still practiced to limit the problem of stray dogs in urban environments (Figure 1).

Several studies have investigated the epidemiological status of rabies in Morocco. We selected the nine that we considered most relevant and which cover the period (1928–2015) (2, 6, 28, 29, 33, 35, 37, 56, 57). Several of the epidemiological characteristics of rabies in Morocco appear stable over the studied period (1928–2015).

#### Rabies Reservoir and Affected Animal Species

Table 2 contains data on the different species involved in animal cases from 1951 to 2015.

**Table 2:** **Animal rabies cases per species from 1951 to 2015**.

Species	1951 (28)^a^		1952 (28)^a^	1953 (28)^a^	1954 (28)^a^		1955 (28)^a^	1956 (28)^a^	1957 (28)^a^		1958 (28)^a^	1964 (33)^a^	1965 (33)^a^	1966 (33)^a^	1967 (33)^a^	1968 (58)		1973–1983 (37)	
Dogs	248		224	193	154		189	191	299		268	291	304	293	236	231		2675	
Cattle	22		38	37	18		33	14	34		16	57	75	11	59	36		1404	
Cats	17		10	6	13		3	5	14		64	19	14	23	20	17		225	
Equids	8		9	6	1		3	5	11		4	10	17	1	5	0		502	
Sheep/goats	3		2	0	0		1	0	2		1	4	4	7	1	3		273	
Camelids	3		1	2	1		2	1	3		1	1	1	1	3	0		15	
Others	0		0	0	0		1	0	0		0	0	0	0	1	0		21	

Total	301		284	244	187		232	216	363		354	382	415	336	325	287		5115	

**Species**	**1997**	**1998**	**1999**	**2000**	**2001**	**2002**	**2003**	**2004**	**2005**	**2006**	**2007**	**2008**	**2009**	**2010**	**2011**	**2012**	**2013**	**2014**	**2015**

Dogs	167	201	194	215	255	192	173	153	106	103	113	100	95	66	64	91	62	64	44
Cattle	98	134	128	133	156	140	148	129	114	113	117	115	106	92	97	149	154	139	128
Cats	21	26	33	33	24	26	14	27	21	16	12	11	12	22	15	12	8	8	9
Equids	67	90	82	79	108	73	85	83	86	83	89	68	58	75	60	74	67	80	48
Sheep/goats	14	21	14	13	26	13	47	28	24	17	22	16	22	9	15	9	16	8	19
Camelids	0	4	3	0	2	0	0	0	0	0	0	0	0	0	0	0	0	0	0
Others	2	0	1	1	1	2	0	5	9	3	2	3	1	5	2	2	3	0	0

Total	369	476	455	474	572	446	467	425	360	335	355	313	294	269	253	337	310	299	248

*^a^Data related to laboratory confirmed animal cases in Casablanca laboratory*.

*Data from 1997 to 2015 provided by the ONSSA Epidemiology and Health Monitoring Service*.

**Figure 2:**
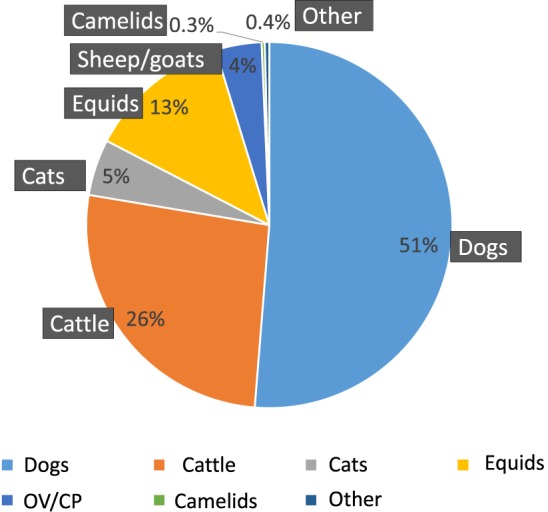
**Animal rabies by species (1951–2015)**.

Since the very first epidemiological studies on rabies in Morocco (2, 29), dogs have been shown to be the reservoir and dog bites the primary source of human contamination. Since 1951, a number of 8254 dogs in all have been diagnosed as rabid, accounting for 51% of all animal notifications (Figure 2). The proportion of dog cases in the total animal rabies in Morocco decreases over the time passing from 82% in 1951 to 20% in 2015 (1951: 82%, 1964: 76%, 1973–1983: 52%, 2000: 45%, 2007: 32%, 2010: 25%, and 2015: 18%). This can be explained by the fact that veterinary services give more attention to rabies diagnosis on livestock because of its economic value (35) and concentrate less on the known vector (i.e., dog).

All the authors agree that herbivores (cattle, sheep, goats, equids, or camelids) are victims. Notifications of rabies in cattle come second to notifications concerning dogs in all the studies (4,244 cases, i.e., 26% of animal rabies cases). Contrary to the tendency evolution in dogs, diagnosis pressure on herbivores (cattle, ovine, goat, horses, and camels) is growing over the time passing from 12% in 1951 to 79% in 2015 (1951: 12%, 1964: 19%; 1973–1983: 43%, 2000: 47%, 2007: 64%, 2010: 65%, and 2015: 79%). This is probably, as previously said, because of economic value of livestock (35) that imposes a close surveillance.

Several studies have emphasized the role of cats in human contamination (21, 33, 35). In all, 800 cats have been notified as rabid and account for 5% of total registered animal rabies cases between 1951 and 2015.

Rats are regularly mentioned in diagnosed cases and even quoted as a rabies reservoir in natural surroundings (33, 37), but since 1980s, they have no longer been named in reports and are classified in the “Others” category of Table 2. Indeed, WHO considers that this species does not play an epidemiological role and is rather an epidemiological dead end (59).

Morocco is the natural environment for some 30 bat species (order *Chiroptera*, families *Rhinopomatidae, Emballonuridae, Nycteridae, Rhinolophidae, Hipposideridae, Vespertilionidae*, and *Molossidae*) (60, 61). To date, as far as we know, rabies has never been detected among these species, and no bats have never been reported as a source of human contamination in Morocco.

#### Seasonality of Rabies

In 1959, Chevrier (28) noted an annual cycle of animal rabies cases, with one peak in the spring and another in the autumn. The link between the cyclical nature of cases and the sexual cycle of bitches was only described in 1985 (35). To date, this seasonal variability is stable.

Several authors (28, 38) have pointed out a cyclical increase in the incidence of rabies every 6–8 years. No explanations have ever been provided for this cycle, which appears to have been broken after the instigation of the first NRCP from 1986 to 1990.

#### Geographical Distribution

No regions of Morocco are free from the disease. Rabies is a rural disease, 80% of notifications originating in the countryside and only 20% in towns. In rural areas, it is most often found in a 30–50 km perimeter around major towns rather than in remote areas of the countryside or mountainous regions where there are fewer people (28). Chevrier’s observations have been confirmed by all the studies that have followed and are still relevant today (6).

The close relationship between the density of the human population and that of the dog population is also highlighted as a risk factor, because the denser the human population, the denser the dog population too and the higher the risk of exposure to rabies (28). The dissemination of the rabies virus along road networks was revealed by Fassi-Fehri et al. (37). These data were later confirmed by molecular biology research in 2010 (62).

**Figure 3:**
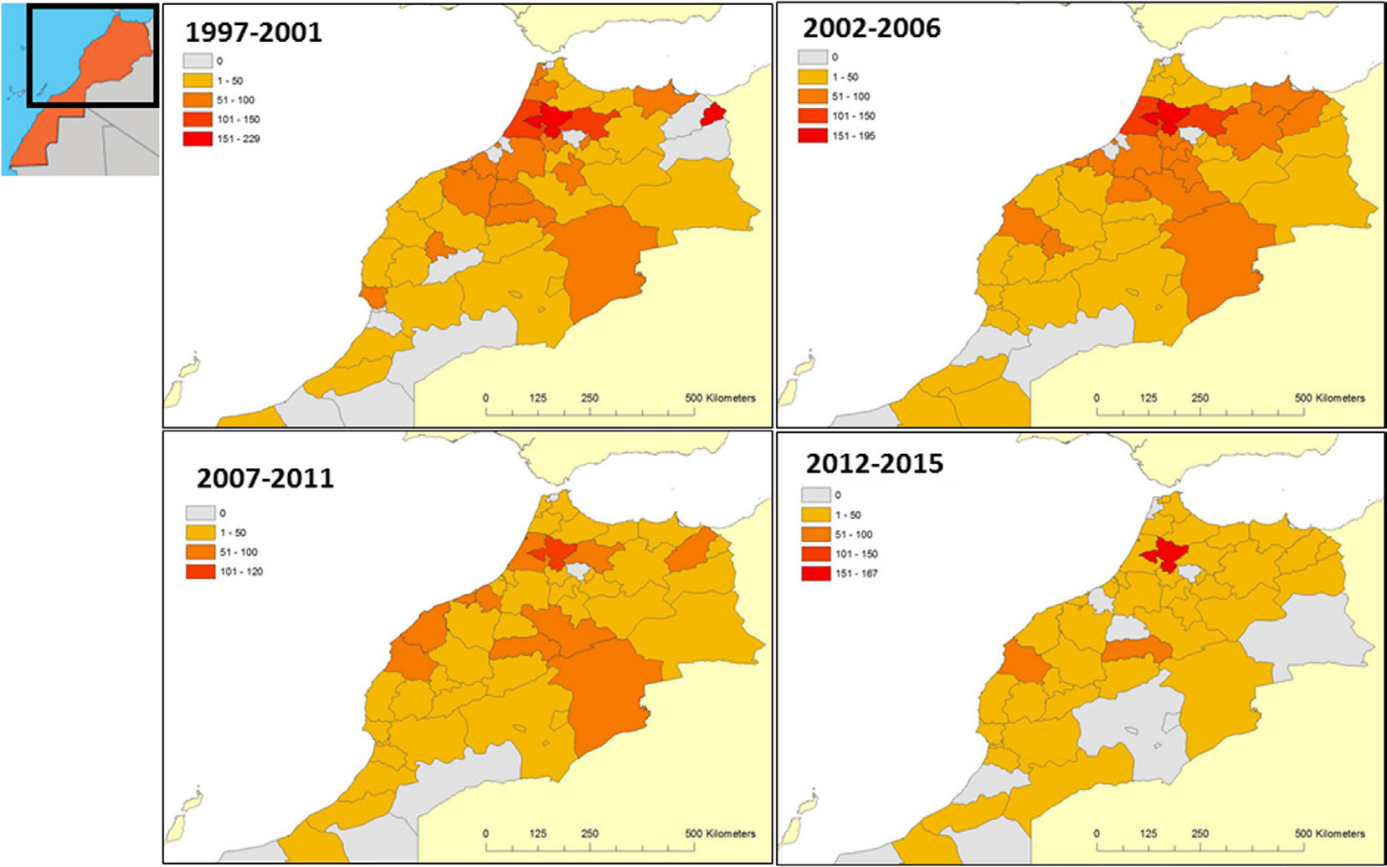
**Geographical distribution of animal rabies in Morocco (1997–2015)**.

Figure 3 shows geographical distribution of animal rabies in Morocco provinces for 1997–2001, 2002–2006, 2007–2011, and 2012–2015. The five most badly affected provinces according to 1976–2015 period data were Sidi Kacem (703 cases), Kenitra (608 cases), Safi (532 cases), Casablanca (492 cases), and Meknes (488 cases) [(35) and ONSSA Epidemiology and Health Monitoring Service]. The geographical distribution of cases shows that rabies is endemic to the whole of Morocco with the exception of desert regions (southern provinces). It may be seen that the distribution of rabies cases is related to the intensity of farming activities, thus confirming that Chevrier’s observation in 1959 (28) is still valid in 2015.

#### Human Rabies

The vaccination of humans following exposure to the rabies virus was first practiced in Morocco by IPT (1). From 1923 to 1932, 361 people in all were treated (21) and from 1951 to 1958, this figure increased to 1,500 people treated on average per year (28) and continued to rise from 3,156 in 1964 to 10,209 in 1973 (36). The number of people treated per year was more or less stable between 1977 and 2003, averaging 13,789 (38, 41, 42, 44–48). From 2004, this figure more than doubled, reaching an average of 29,163 people from 2004 to 2015 (data provided by the Epidemiology and Disease Control Directorate of the Moroccan Ministry of Health). The human rabies is a notifiable disease in Morocco since 1967 (63).

**Figure 4:**
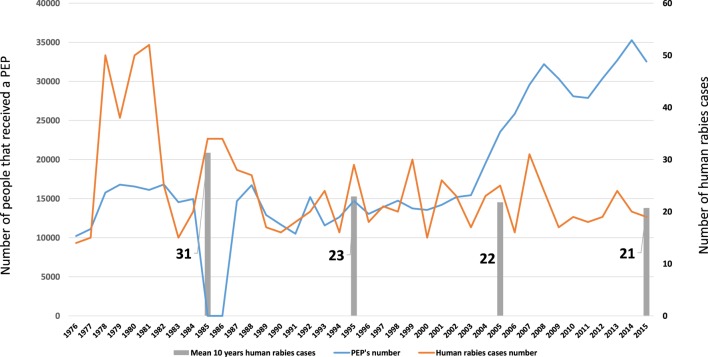
**Number of human rabies cases and number of people who received postexposure prophylaxis (PEP) (1964–2015)**.

Table 1 and Figure 4 present the annual number of human rabies cases and the number of persons who received PEP from 1971 to 2015. The number of human rabies cases is slightly decreasing over time: the annual mean of human cases for 1976 to 1985 is 31 (min = 14 and max = 52), dropping to 23 (min = 16 and max = 34) for 1986 to 1995, then 22 (min = 15 and max = 30) for 1996 to 2005, and finally 21 (min = 16 and max = 24) for 2006 to 2015. The number of persons who received PEP increased in the same time: from 1986 to 2005, there were 14,633 PEPs per year on average (min = 10,496 and max = 23,564), but this figure has more than doubled, reaching an annual mean of 30,687 (min = 25,857 and max = 35,279) for the period from 2006 to 2015. This is no doubt thanks to the efforts of local communes which, under the impetus of the Ministry of the Interior (64), have opened new rabies treatment centers, whose numbers have risen from 120 in 2008 to 147 in 2012 (56, 63).

Up to now, Morocco has applied the 2-1-1 protocol for PEPs by the intramuscular route (63). This could increase by 60–80% the number of people treated for the same budget in rural areas if it used the intradermal delivery of fractioned doses recommended by WHO (65). It has been proven that poor people often do not spend time and money traveling to receive PEP (66).

Generally speaking, human rabies in Morocco matches the geographical distribution of animal rabies (35). Dogs are the main species responsible for human contamination (91% in 1935, 80% in 1965, 95% in 1973, and 80% in 1985) (2, 30, 35, 37). The frequency of contamination is greater among the male population (around 80%) and among young people (around 40% among children less than 15 years old). Bites often involve the legs (around 45%), face (around 15%), and hands (around 35%), all other locations (such as the neck or genitals) being rarer (35, 37, 38).

Although, as previously said, the number of human rabies cases of rabies is decreasing, it should be remembered that official notifications are generally underestimated (13) and that they may only concern hospital cases (37). There is a need to investigate the origins of underreporting and give feedback after detailed analysis over several years of the files on human deaths. This would reveal weaknesses in the system and possible means of improvement. One of the reasons of underreporting could be the population’s refusal to allow an autopsy of their loved ones for cultural reasons. Alternatives to autopsy by non-invasive methods such as skin biopsies or supraorbital sampling could lead to an increase in the number of rabies cases reported and provide the diagnostic laboratory with precious samples (66). A report following a 2-week WHO expertise carried out in 2001 in different places of Morocco (67) as well as a study (39) revealed that human deaths are linked to an inadequate prophylaxis and suggest an improvement of training of physicians in antirabic centers.

Morocco has one diagnostic laboratory for human rabies, which only uses postmortem analytical methods. It confirms about 30% of clinical cases (63). The use of quick new reliable tests (lateral flow or immunohistochemical methods) in local, non-specialized laboratories (66) could help increase the rate of laboratory confirmations.

#### Genetic Variability of the Rabies Virus

Since the rabies virus was first sequenced in 1995, the Moroccan strains have been identified as belonging to genotype Africa 1 (68). The RabMed Control project included a phylogenetic study of 133 samples of canine rabies from 28 Moroccan towns between 2004 and 2008 (62). This study revealed:

- that rabies transmission in Morocco does not correspond to a rabies virus transmission model for a wild canine population;
- that the spatial dynamics of the rabies virus in Morocco is better described by mean road distance between outbreaks (51 km, with a minimum of 34 and maximum of 72 km per year), suggesting human intervention in the transmission of the rabies virus through the movement of rabid dogs (62).

### National Rabies Control Plans

Since 1980s, several different NRCPs have followed in succession:

– *NRCP covering 1986–1990*: plan drawn up as part of the WHO rabies control program in Maghreb countries (3, 4). An interministerial committee was set up to draft a NRCP, whose broad lines included:
  ◦ Raising awareness among the public and providing health education (causes, seriousness and consequences of rabies, reasons for and scope of the control measures proposed).
  ◦ Health prophylaxis: eliminating stray domestic carnivores through stray dog culling campaigns. Reducing the availability of food for stray domestic carnivores. Monitoring suspected and biting animals. Checking the movements of wild and domestic carnivores. Making diagnosis of animal rabies regional rather than national.
  ◦ Medical prophylaxis: targeted vaccination of 80% of 700,000 dogs (this being the estimated number of owned dogs at that time) with a stable, low-cost vaccine already proven to be harmless. A government veterinary vaccine company named Biopharma developed an inactivated rabies vaccine for animal use by the parenteral route in the framework of the plan (35). The maximum number of dogs vaccinated under this plan was in reality 229,231 in 1989 (5).
– *Amendment of 1993*: actions focused on vaccinating owned dogs (325,780 dogs vaccinated in 1994), while reducing the culling of stray dogs to the necessary minimum as this measure leads to an increase in the rate of replenishment of the dog population and thus an increase in the proportion of young unvaccinated dogs, not to mention its negative impact on the participation of owners in vaccination campaigns. This explains the need to limit culling to “true stray dogs” (i.e., feral dogs).
  As the plan progressed, this component was not strictly and constantly applied. From 1991 to 1992, the general rabies vaccination campaigns were not carried out because this period coincided with the outbreak of both African horse sickness and foot-and-mouth disease, which mobilized substantial human and material resources (Figure 1). From 1996 to 1998, the rabies vaccination of dogs was limited to campaigns isolated both geographically and over time (5). Consequently, the results fell well below the vaccination coverage targets set by the NRCP (i.e., 70–80% of the dog population).
– *NRCP covering 2000–2010*: this plan aimed to reduce the number of rabies cases within 5 years. The longer-term objective was to eliminate rabies within 10 years and ensure that the country then remained rabies-free. The strategy proposed, which is based on a generalization of medical prophylaxis supported by targeted health prophylaxis, required a change in regulations, regular awareness-raising activities among the general public, training, and retraining of the main players and the setting up of a national epidemiological surveillance network.

Finally, a system to assess the proposed strategy was set up (5). This plan led to the vaccination of a maximum of 450,917 dogs in 2005 (5). The apparition of various health crises in the livestock sector led to veterinary services control efforts to combat or prevent the diseases by intensifying surveillance and/or vaccination campaigns (sheep pox vaccination campaigns in 2002, 2004, and 2006, avian influenza surveillance in 2005 and 2006, bluetongue vaccination campaign in 2007, and PPR vaccination campaigns in 2008, 2009, and 2010) (34). This prevented the plan from reaching its objectives.

Despite the limited effect of these different plans on the status of rabies in general, they nonetheless laid the foundation for future actions:

– Creation of an interministerial rabies control committee.
– The local production of an inactivated rabies vaccine for veterinary use produced on cells (69).
– Initiation of an operational epidemiological surveillance system (5).
– Development of a laboratory network comprising seven regional laboratories able to diagnose rabies using different OIE reference techniques (immunofluorescence, cell or mouse inoculation, and molecular biology). Of these, three (at Fes, Marrakech, and Rabat) accredited their rabies diagnosis analyses to standard ISO 17025 in 2012. Table 3 shows the number of samples of animal rabies analyzed by laboratories between 1932 and 2015, data concerning the periods from 1932 to 1934, 1952 to 1958, 1963 to 1967, and 2010 to 2015. It may be seen that the mean percentage of samples confirmed positive by laboratory analysis has increased over time from 26% (minimum 20%, maximum 29%) for 1932–1934 to 62% (minimum 52%, maximum 74%) between 1952 and 1958, 70% (minimum 57%, maximum 86%) for 1963–1967 up to 104% (minimum 70%, maximum 168%) for the 2010–2015 period. Several rates exceed 100% for the latter period, probably due to the fact that several samples sent to a laboratory concern the same rabies outbreak. Nonetheless, an average of 20% (minimum 12%, maximum 30%) of cases were reported following clinical signs but not confirmed by a laboratory during the 2012–2015 period, which goes against OIE recommendations (70).

**Table 3:** **Number of declared animal rabies cases and number of animal rabies laboratory-confirmed cases from 1932 to 2015**.

	Year (reference)										
	1932 (2)	1933 (2)	1934 (2)	1952 (28)	1953 (28)	1954 (28)	1955 (28)	1956 (28)	1957 (28)	1958 (28)	1963 (33)
Official declaration	80	91	129	452	390	334	381	398	563	479	493
Laboratory confirmed rabies cases	23	18	38	284	243	185	232	207	363	354	365
Percentage	29	20	29	63	62	55	61	52	64	74	74

		**1964 (33)**	**1965 (33)**	**1966 (33)**	**1967 (33)**	**2010 (50)**	**2011 (51)**	**2012 (52)**	**2013 (53)**	**2014 (54)**	**2015 (55)**

		446	663	651	–	269	253	337	310	299	248
		382	405	374	325	453	346	297	243	210	204
		86	61	57	–	168	137	88	78	70	82

Since 2014, WHO, OIE, and FAO—supported by the Institut Pasteur network—have launched an initiative to eradicate rabies in North Africa in general, and Morocco in particular, by around 2020. This initiative is supposed to materialize the implementation of the concept (71). Since the initial kickoff workshop in 2014, the actions in the framework of this initiative have not yet been communicated.

The parenteral vaccination of owned dogs in rural settings has been the key measure in rabies control programs in Morocco since 1911 (1). However, this measure was rapidly revealed to be insufficient in the light of the tiny proportion of dogs vaccinated compared to the size of the total dog population. The culling of dogs as a means of controlling the dog population was suggested in 1935 (2). This twofold mechanism, involving the vaccination of dogs and culling of stray dogs, is still carried out today in Morocco.

Several reasons for the failure of this strategy have been identified from 1938 on and are still relevant today:

– Vaccinating dogs against rabies has a moral and sentimental rather than economic aspect to it compared to other livestock vaccinations. Vaccinating dogs is considered a luxury (29).
– The culling of stray dogs is not enough and only affects a few isolated cases. The mobility of stray dogs and their relations with the unvaccinated dogs found in douars (tent villages) are too frequent to allow this measure to fully succeed (28).
– By religious conviction, Moroccans refuse to destroy life, which explains why the dog population is so big (28).
– Moroccan farmers usually know the fatal issue of bite-related street rabies, yet refuse to sacrifice infected dogs by negligence or superstition (33).
– The rabies control program in terms of medical and health measures is insufficient (6).
– There are many players and they do not work closely enough together (6).
– There are insufficient resources (6).
– The socioecology of dogs is not yet well known (6).
– The management of rubbish dumps has to be rethought for the whole of the kingdom (6).
– Dog owners are not sufficiently aware of the problem and their responsibilities (6).

Several judicious proposals have been put forward by various authors from 1935 on to improve the situation and are still relevant:

– Vaccinate community dogs that roam free (2).
– Inform dog owners as actively as for other livestock vaccinations (2).
– Think of possible actions by kennel clubs, animal protection services, and even public hygiene services (2).
– Limit the number of dogs in douars (28).
– Instigate mandatory registration and vaccination of pet dogs (28).
– Investigate canine socioecology (56).
– Study the feasibility of oral vaccination as a complement to parenteral vaccination (6, 56).
– Regularly educate and raise awareness of rabies among the general public (6, 28).
– Associate/involve communes in the combat to control rabies (6).
– Strengthen intersectoral cooperation and give fresh impetus to the provincial rabies control centers (6).
– Improve infrastructures: rural slaughterhouses and public rubbish dumps (6).

### Moroccan Dog Population

Several authors (5, 13, 72–74) have underlined the importance of a good estimation of the dog population in the success of rabies control programs. According to WHO, the size of the dog population may be estimated through the human:dog ratio (59). Studies of the dog population carried out in Morocco in 1993 assessed the human:dog ratio at 5.93 in rural settings and 25.36 in urban settings. In 1999, the ratio was found to be 7.93 in rural settings and 80.94 in urban settings (5). More recently in 2013, estimations of this ratio for North Africa gave 3.84 in rural settings and 9.83 in urban settings (73) and for Africa in general, 7.40 in rural settings and 21.20 in urban settings (13).

To estimate the current dog population, we chose the mean of national dog population estimates obtained in 1990s and international estimates obtained in 2005 and 2013, giving a human:dog ratio of 6.14 in rural settings and 34.33 in urban settings. This value is no doubt biased but the bias is acceptable. By applying the calculated human:dog ratio and taking into account Moroccan demographic data (the Moroccan population being estimated in 2015 at 34,271,622 in all, including approximately 13,458,258 in rural settings and 20,813,364 in urban settings) (data from target populations of health programs. Health Ministry website http://www.sante.gov.ma), we may conclude that the dog population is estimate to stand at 2,798,126 (of which 2,191,930 in rural settings and 606,195 in urban settings). This number could be overestimated but we consider that it is better to overestimate the target population rather to underestimate it.

It should be noted that generally speaking, there are few data about Moroccan dog population features (sex ratio, mean age, and life expectancy of an owned or stray dog). Although Moroccan legislation requires dog owners to vaccinate their pets, it remains silent on the management of this population, as mentioned earlier. Neither does it inspire responsible ownership in order to prevent dogs creating a nuisance within the community.

Managing the dog population is a complementary measure in rabies control and reducing the dog population does not directly affect the transmissibility rate (R0, i.e., the reproduction number) of the rabies virus (75). This runs contrary to the idea that canine rabies can be eliminated by reducing the density of the dog population (59, 66). This reduction in density is frequently achieved through culling campaigns that often prove counterproductive and have a major impact on animal welfare (11). Indeed, dog owners frequently begin hiding their dogs, moving them to other areas, or indeed acquiring new dogs to replace those killed. This behavior is linked to the need for dogs, itself related to their function. In Morocco, dogs are mainly used as guard dogs, to watch over livestock (31%), a combination of both (87%) or to hunt (2%) (76). Dog movements help disseminate the rabies virus, as reported in Indonesia (75) and North Africa, including Morocco (62). Furthermore, dogs that are easy to vaccinate are those that are the easiest to cull, thus reducing the dog population’s immunity (66).

Dog sterilization campaigns are a key tool in the management of unwanted dogs. They reduce the trouble caused by dogs, along with their aggressive behavior and thus dog bites. They improve the human population’s acceptance of free-roaming dogs and improve the health and life expectancy of dogs in a roaming population (77). Sterilization would increase the effectiveness of vaccination campaigns by stabilizing the size of the dog population and reducing its renewal rate. It is an important complementary measure in a rabies control program whose key thrust is vaccination coverage (66).

Currently, Moroccan communes are responsible for taking measures to prevent animals from roaming free. They must collect, check, and impound stray dogs in compliance with article 50 of the Communal Charter (64).

However, these functions are not fulfilled in most towns and those communes where they have developed various systems:

– Some communes organize armed hunts with hunting associations following citizens’ complaints about the number of stray dogs. These hunting parties are held in daytime in urban settings. Communities and the press are increasingly hostile to this system (78, 79).
– Other communes lay strychnine-poisoned baits. This very dangerous method is illegal because the use of strychnine is regulated by Moroccan law (80). Furthermore, the use of this poison has a major ecological impact due to the harm it can cause to both wildlife and groundwater.
– Communes organize the capture of dogs in cooperation with the town’s veterinary services or animal defense organizations. The veterinary services then put the animals down humanely using barbiturates (81).

There are several possible ways of getting out of this situation, alone or in conjunction depending on local conditions:

– Stimulate the networking of animal protection associations through contract programs (82). Several animal welfare associations are active in different regions of Morocco, the oldest having been active since 1916 (83–90). One example is an association working in the town of Essaouira that captures, identifies, sterilizes, and vaccinates against rabies then frees the stray cats and dogs captured at the place they were found (91). This model could be followed and extended to other towns in order to manage the stray cat and dog population. The Department of Agriculture is very experienced in monitoring and implementing contract programs with professional associations (92), an experience that could be capitalized on to develop contract programs with animal protection associations in order to manage the stray cat and dog population while still complying with departmental prerogatives and respecting animal welfare criteria.
– Delegate the management of municipal pounds and the impounding of stray dogs to the private sector. The experience of Madrid, Spain, in this area has recently been presented to the elected officials of Khemisset in the framework of the international partnership between Morocco and Spain on hygiene and public health (93).
– Raise awareness of dog owners about what being a responsible owner involves, especially with respect to the future of puppies from unwanted litters. This suggestion had already been put forward in 1959 (28).
– Use chemical or hormonal sterilization in conjunction with rabies vaccinations (94–97). It would be very useful to stimulate research into this possibility in the Moroccan context.

### Vaccination Coverage of the Dog Population

The most efficient means of preventing human rabies cases is to reach a 70% vaccination coverage of the dog population (12, 66). The vaccination coverage rate of the dog population in Morocco can be estimated by dividing the total number of vaccinated dogs by the total dog population, calculated by adding together the following two figures:

– The number of dogs vaccinated in rural settings, which corresponds to the number of dogs vaccinated during free mass vaccination campaigns organized by the government. The rural dog population represents 78% of the total dog population, as previously estimated in this study.
– The number of dogs vaccinated in urban settings, which shall be estimated using the number of rabies vaccines sold by the veterinarian pharmaceutical companies authorized in Morocco to private veterinarians, divided by two. This is because the government-organized mass vaccination campaigns are not held in urban settings, and a dog living in town will only be vaccinated if its owner has it vaccinated by a private veterinarian. In practice, owners do not all follow the same vaccination protocol for their dog, despite the indications of vaccine manufacturers and private veterinarians. An owner may take his dog to the veterinarian only once for the primo-vaccination, or twice for a primo-vaccination divided into two injections, or take his pet for its annual booster only once in its life or never again after the primo-vaccination (personal observation). We consider that with a bias we accept that an owned dog in an urban setting is injected with rabies vaccine on average twice during its life. So the number of town-dwelling dogs, therefore, corresponds to the number of doses of rabies vaccines sold divided by two. The rabies vaccine is sold to private veterinarians by seven veterinary establishments. The list of products authorized per company is available on the ONSSA website (98). The vaccines marketed in Morocco are all inactivated adjuvanted vaccines prepared using different strains (VP12, G52, PV, Flury LEP, VP13, and CVS). The number of doses sold annually from 2009 to 2015 was provided by the companies themselves (Table 4). The number of sold doses increases by almost 5% each year.

**Table 4:** **Number of rabies vaccine doses sold to veterinarians in the private sector from 2009 to 2015**.

Year	Number of doses
2009	68,530
2010	71,040
2011	86,920
2012	70,200
2013	86,660
2014	85,640
2015	100,020

It should be noted that the vaccination coverage from 2009 to 2015 never exceeded 6% of the total dog population. Urban vaccination coverage fluctuates between 6.51 and 8.25%, while rural vaccination coverage fluctuates between 2.47 and 5.73% for the same period. The highest coverage rate in a rural setting was obtained during 2001–2010 NRCP, with 20.64% in 2005. The other national plans resulted in a maximum of 15.79% in 1994 and 11.41% in 1989. The data for urban vaccination coverage are not available before 2009. In any case, whether urban, rural, or total, the vaccination coverage rates are too low to break the transmission cycle of rabies virus. These data serve to confirm that since the first canine rabies vaccination in 1928, Morocco has only managed to vaccinate a tiny part of its dog population and is a very long way off the 70% vaccination coverage recommended by international organizations OIE and WHO (59, 70). Chevrier’s observation in 1965 that “this vaccination is of no prophylactic benefit compared to the total dog population, so the issue needs to be completely reviewed” is just as true today (30). This operation has always been carried out with the same strategy, using government officials for vaccinations in rural areas and private veterinarians in towns. In addition, all previous official rabies control plans have always targeted to vaccinate owned dogs population rather than targeting the whole sensitive population (i.e., total dog population) and those plans did not propose any strategy to vaccinate the stray dogs. This led to the described limited results of vaccination coverage and rabies control failure.

In our opinion, one of the ways of improving canine vaccination coverage would be to involve private veterinarians in the free government-run vaccination campaigns, whether in rural or urban settings, as part of a public–private partnership. The synergy between ONSSA and private veterinarians during an action program to vaccinate sheep and cattle was successful in tackling various livestock diseases (PPR vaccination campaigns in 2008, 2009, and 2010; bluetongue vaccination campaign in 2007; sheep pox vaccination campaigns in 2011 and 2013, and foot-and-mouth disease vaccination campaign in 2015) (34). This public–private partnership managed on more than one occasion to successfully vaccinate 19.5 million sheep and 3.2 million cattle (92) within 3–4 months, resulting in a vaccination coverage of about 97% (34). The pathway to participation of private veterinarians has already been cleared through a decree establishing government remuneration of veterinarians in the private sector for the vaccination of dogs and cats in January 2015 (99).

Oral vaccination is another promising possibility currently under investigation for dogs. WHO has proposed various application scenarios, such as door-to-door vaccination, distribution at a central point, or a wildlife model (100). The latter involves jettisoning baits from aircraft, which is not very practicable for the oral vaccination of dogs. Unlike wildlife, the dog’s habitat is closely linked to that of humans, making aerial distribution such as practiced in un- or little-populated areas where wildlife live unrealistic for dogs. Unlike injectable rabies vaccines, which are inactivated and often contain adjuvants, oral rabies vaccines are live attenuated vaccines or recombinant protein vaccines. Several oral rabies vaccines have market authorizations, yet only two are considered sufficiently safe by WHO (59, 100) to be used in proximity to people. One is Rabigen SAG2™, produced by Virbac SA, France (101), and the other is Raboral V-RG^®^, produced by Merial Inc., USA (102). The other commercial vaccines have residual virulence that can induce rabies, in target and non-target species (103, 104).

Studies have been carried out on the use of V-RG on dogs in Morocco, investigating the appetence of the bait and its efficacy under controlled conditions, but the study results were limited (49, 105). This had already been observed by other authors (72). Further research would be beneficial to investigate the efficacy of this vaccine on dogs and cats.

The results of a pilot study carried out in Morocco on the use of SAG2 for oral vaccination of dogs in field conditions were promising. The dog population in the study zone was composed of 70% of owned dogs and 30% of stray dogs. Using the door-to-door model (59), 77% of owned dogs ate the bait. Using the wildlife immunization model (59) in stray dogs, up to 73% of baits disappeared and 68% of the capsules containing the SAG2 vaccine were found pierced (106). Further research is needed to fine-tune the distribution strategy before large application in the field.

In our opinion, the oral vaccination of dogs is a way forward in conjunction with parenteral vaccination as recommended by WHO (59). It would reach stray dogs or dogs that are inaccessible due to their aggressive behavior and could be organized during mass parenteral vaccination campaigns. It would also be possible to distribute baits to well-informed owners so that they themselves give their dog the bait (on the condition that is in individual wrapping and kept at 4°C for no more than a few days), with strict instructions on giving the bait as soon as it has been opened. If not consumed straight away, it must be destroyed.

The use of new technologies has given good results by allowing real-time monitoring of mass vaccination campaigns (107). Several parameters can be assessed in real time, such as the number of dogs vaccinated, the proportion of dogs vaccinated compared to the total population, the GPS position of vaccination locations, and a map of the vaccination coverage per region for the whole country (107, 108). This should be able to be used in Morocco, one of the main advantages being that it would enable resources and vaccination teams to be redeployed in real time without having to wait for the end of a campaign before drawing conclusions.

All these difficulties underline the peculiarities of rabies compared to other livestock diseases, where often just one vaccination campaign is enough to improve the situation. For rabies, substantial efforts have to be made over several consecutive years on a sufficient large range of dog population, each interruption losing the benefits accrued up to then. This shows the importance of political determination and coordinated efforts. Rabies should never be accepted as banal. Even though it is not a disease with a direct economic impact (dogs generally not having an “economic” value), political efforts must be kept up over a long period so that the enthusiasm of vaccination teams does not wane year after year. Vaccination initiatives must be maintained in the light of an ever-increasing dog population and efforts should not be allowed to be diverted to other health priorities.

### Application of the “One World, One Health” Concept

The “One World, One Health” concept was initiated in 2008 by FAO, OIE, and WHO with the support of the United Nations Children’s Fund, the United Nations System Influenza Coordination, and the World Bank (109). It aims to develop a joint strategic network for coordinating medical, veterinarian, and environmental health policies to face up to the risks associated with the emergence or reemergence of zoonoses (109, 110). The major advantage of this concept lies in the economies of scale afforded through the efficiency of control and elimination measures, which is why it is so advantageous for low-income nations (66).

Latin America is a successful example of the “One World, One Health” concept as regards rabies. The strong political will of all the countries in that region was channeled into national public health and animal health action plans. Mass dog vaccination campaigns were held (59). The cooperation with NGOs, animal welfare organizations, and a close public–private partnership supported by efficient communication with local communities which were also heavily involved were the keys to the success of this rabies elimination program (66). Results were visible after a few years, by which time animal and human rabies cases had dropped by 90% (59).

The bases for this “One World, One Health” approach already exist in Morocco. The interministerial committee on rabies control is just one proof. The action of this committee should be supported by new legislation that gives it broader powers. Kenya, for example, created a “One Health Office” in 2011 in order to develop control strategies for the country’s priority zoonoses, including rabies (111).

Since 2009, Morocco has celebrated World Rabies Day (112), an initiative launched in 2007 by the Global Alliance for Rabies Control. Held on September 28 each year, World Rabies Day seeks to focus the attention of the international community on rabies prevention and control measures (113). The World Rabies Day events from 2009 to 2013 were organized jointly by the National Food Safety Office, ONSSA, and the Hassan II Agronomics and Veterinary Institute, with the participation of other ministerial departments such as the Moroccan Ministry of Health, the Ministry of the Interior, the Ministry of National Education, and the Ministry of Islamic Affairs, in addition to local authorities, the Moroccan Association of Veterinary Professors–Researchers, the Society for the Protection of Animals and Nature along with the media. Various rabies-related actions were held on these occasions, including a rabies science day, the vaccination of dogs, information on rabies targeting elected officials in the communes, Friday sermon on rabies throughout all the mosques in Morocco, educating and informing primary school children about rabies, awarding of a prize to the journalist having done the best report on rabies, a radio broadcast, and inclusion on the news of the main TV channel (112). All these local and national events have raised awareness among the general public, especially young people, and will bear fruit in the long term.

## Conclusion

Despite the fact that the first rabies vaccination for dogs dates back to 1911 in Morocco, rabies is still endemic. Despite an annual decline in the number of cases, the situation is alarming, and 19 deaths by rabies registered in 2015 is too high a price to pay. The epidemiological characteristics of the disease have been stable for the past century: dogs remain the vector and the reservoir of the disease, while other animal species are its victims. There are two peaks in the number of cases each year: one in the autumn and the other in the spring, in keeping with the sexual cycle of bitches. Rabies is endemic throughout Morocco, with a higher concentration of cases in areas where the human population is denser. The disease is mainly rural. The circulating virus is genetically homogeneous and belongs to the Africa 1 genotype. Several rabies control plans have been implemented, all with the same strategy of vaccinating owned dogs, opening rabies treatment centers, and increasing the budget for PEP. Unfortunately, the results are limited.

Morocco did not develop any strategy for dog population management, either owned dogs or stray dogs. Only a few old data exist about dog population characteristics. The Moroccan legislation does not deal with the concept of responsible dog owner, and rabies control strategies need to be developed and tested with all stakeholders’ contributions to control and stabilize dog population.

In our opinion, the major reasons of failure of Moroccan strategies to control rabies are as follows:

– Lack of dog population knowledge and control
– Failure to implement any rabies control strategy in the event of another livestock health crisis
– Problem of human rabies case management
– Lack of coordination between departments
– Lack of law enforcement especially regarding antirabies dog vaccination
– Absence of the concept of dog responsible owner

Morocco needs a new approach to rabies control based on scientific advances and success stories in other parts of the world. Such an approach should include the following elements:

– Implementing an integrated approach in keeping with the “one world, one health” concept,
– Using a public–private partnership to extend the vaccination coverage of dogs and manage dog pounds,
– Updating current legislation to include:
  ◦ the notion of a responsible owner;
  ◦ sanctions if the law is not applied.
– Initiating contract programs with animal protection associations to sterilize dogs,
– Managing the dog population while complying with human population needs and animal welfare principles,
– Studying the socioecology of dogs (size and structure of the dog population) to fine-tune control strategies to be routinely implemented before each campaign,
– Combining parenteral and oral vaccination in preselected areas,
– Using new technologies to disseminate information better, including awareness of the general public,
– Improving and updating knowledge of professionals involved in rabies prevention and control by organizing regular trainings,
– Drafting and assessing the strategy chosen using international models,
– Estimating the annual economic cost of rabies (animal control and human prevention)

Rabies is not a fatality with which we have to live, but a fully preventable disease. Each day spent considering and refining strategies is another day of needless deaths.

## Author Contributions

SD, FC, MB, and OF-F: substantial contributions to the conception or design of the work; or the acquisition, analysis, or interpretation of data for the work; and drafting the work or revising it critically for important intellectual content; and final approval of the version to be published; and agreement to be accountable for all aspects of the work in ensuring that questions related to the accuracy or integrity of any part of the work are appropriately investigated and resolved. MW, ER, and NA: substantial contributions to the conception or design of the work; or the acquisition, analysis, or interpretation of data for the work and drafting the work or revising it critically for important intellectual content.

## Conflict of Interest Statement

The authors declare that the research was conducted in the absence of any commercial or financial relationships that could be construed as a potential conflict of interest.

## Footnotes

^1^http://www.sgg.gov.ma.

^2^www.onssa.gov.ma.
